# Cloning, Purification, and Partial Characterization of the *Halobacterium sp.* NRC-1 Minichromosome Maintenance (MCM) Helicase

**DOI:** 10.2174/1874285800802010013

**Published:** 2008-03-12

**Authors:** Nozomi Sakakibara, Mimi Han, Claire R Rollor, Rebecca C Gilson, Courtney Busch, Gunyoung Heo, Zvi Kelman

**Affiliations:** 1 University of Maryland Biotechnology Institute, Center for Advanced Research in Biotechnology, 9600 Gudelsky Drive, Rockville, MD 20850, USA; 2 Department of Cell Biology and Molecular Genetics, University of Maryland College Park, College Park MD 20742, USA

## Abstract

The MCM gene from the archaeon Halobacterium, with and without its intein, was cloned into an *Escherichia coli* expression vector, overexpressed and the protein was purified and antibodies were generated. The antibodies were used to demonstrate that *in vivo* only the processed enzyme, without the intein, could be detected.

## INTRODUCTION

Chromosomal DNA replication is an essential process for all organisms. It insures the accurate duplication of the genetic information prior to cell division. The process is highly controlled and coordinated, as aberrant DNA replication can lead to malignancies and cancer. The process is conserved in all organisms including bacteria, archaea, eukarya, viruses, and bacteriophages.

*Halobacterium *sp. NRC-1 is a halophilic archaeon with an optimal growth temperature of 42ºC and a 2 hr generation time [1]. The genome of *Halobacterium *sp. NRC-1consists of 2.5 Mb and encodes for about 2,600 proteins. The entire genome consists of one large chromosome (2 Mb) and two extrachromosomal replicons, pNRC100 (191 kb) and pNRC200 (365 kb).

The minichromosome maintenance (MCM) complex is thought to function as the replicative helicase of archaea and eukarya [2,3]. One MCM homologue has been identified in the genome of *Halobacterium*. In contrast to other archaeal MCM proteins studied to date, the *Halobacterium *protein contains an intein.

In this study we describe the isolation, purification and *in vivo* identification of the single MCM helicase from *Halobacterium *sp. NRC-1. It was found that *in vivo* only the mature enzyme, from which the intein has been removed, could be identified.

## MATERIALS AND METHODS

### Sequence Alignment of MCM Proteins

The Halobacterium sp. NRC-1 MCM protein sequence was aligned with four other archaeal MCM proteins (*Methanothermobacter thermautotrophicus*, *Thermoplasma acidophilum*, *Sulfolobus solfataricus *and* Archaeoglobus fulgidus*) using the MUSCLE algorithm (http://www.ebi.ac.uk/muscle/).

### Cloning the MCM Gene

The Halobacterium sp. NRC-1 MCM gene was amplified using PCR from genomic DNA (kindly provided by Brian Berquist). The PCR reaction was performed with 100 ng genomic DNA, 200 µM dNTPs, 5 units of OptimaseTM polymerase (Transgenomic), 1 x Optimase reaction buffer, 1.5 mM MgSO4, 10% DMSO and 0.4 µM of primers, N101 (5’-CCGCTCGAGGCTAGCCATATGGATCCGGACCTGGC-CGACGATTACATCAGCC-3’) and N102 (5’-CCG-CTCGAGCCATGGCTAGATCGAGCGCAAGTGGTCCG-TGTTC-3’), where the XhoI restriction site is underlined, and the NdeI site shown in bold type. Twenty PCR cycles were performed as follows: denaturation at 95ºC for 2 min, annealing at 70ºC for 45 seconds, and elongation at 72ºC for 5 minutes. The PCR product was purified using the QIAquick PCR purification kit (Qiagen), digested with XhoI and ligated into the XhoI site of pBluescript KS- (Stratagene). The sequence was confirmed by sequencing and the clone was designated pBS-MCM. The gene encoding MCM was excised from the pBS-MCM vector by cleavage at the NdeI and XhoI sties and cloned into pET-16b vector (Novagene). This clone is designated pET-MCM.

To generate the MCM gene construct without the intein, a PCR-based method was used [4] using pBS-MCM as template. The PCR reactions were performed as described above with the following two primer sets to amplify the two exteins. One set was N101 together with 5’- GGGGCGCGA-TGTTCTGCACGTAGGAGATCATCTGGGACTTCCCGG-TTCCCGGGTCACC–3’ and the other set was N102 together with 5’-GGTGACCCGGGAACCGGGAAGTCCCAG-ATGATCTCCTACGTGCAGAACATCGCGCCCC-3’. Two exteins encoding PCR products were purified using the QIAquick purification kit and used as template (10 ng each) for PCR amplification using N101 and N102 as primers. The resulting PCR product contained the MCM gene minus the intein. This product was cloned into pET-16b, sequenced, and designated pET-MCMint.

### Purification of the MCM Proteins

Plasmids containing the genes encoding for the full-length MCM and the protein without intein were transformed into Codon plus *Escherichia coli* cells (Stratagene). Cells were grown in LB media at 37ºC. When the OD_600_ reached 0.6, protein expression was induced by the addition of IPTG (1 mM final concentration) and the cells were grown for an additional 4 hours. Cells were harvested and stored at -80ºC. Protein purification was carried out at 4ºC as follows. Cells were thawed on ice in lysis buffer containing 10 mM imidazole, 3 M NaCl, 0.5 M KCl, 20 mM Tris-HCl (pH 7.6) and 20% glycerol and disrupted by sonication. The lysate was centrifuged for 15 min at 15,000 rpm in JA-17 rotor (Beckman). The pellet was kept and the supernatant was incubated with Ni-column resin for 1 hr with gentle shaking. Following incubation, the resin was poured into a column and washed with lysis buffer containing 10 mM imidazol. The MCM protein was step eluted with 50, 100, 200 and 300 mM of imidazol.

Since only a small fraction of the induced MCM protein was found in the soluble fraction, the protein was also purified from the pellet using denaturing conditions in urea. The cell pellet was resuspended in buffer containing 8M urea and 20 mM Tris-HCl (pH 7.6) followed by centrifugation for 15 min at 15,000 rpm in a JA-17 rotor. The supernatant was incubated with Ni-column resin for 1 hr with gentle shaking. Following incubation, the resin was poured into a column and washed with lysis buffer containing 10 mM imidazol. The MCM protein was eluted using step elution in lysis buffer containing 6 M urea and 50, 100, and 300 mM imidazole. The fraction with the highest MCM concentration (300 mM imidazol) was dialyzed in buffer containing 20 mM Tris-HCl (pH 7.6), 3 M NaCl, 0.5 M KCl, and 20% glycerol. The proteins were flash frozen in liquid nitrogen and kept at -80^o^C.

### Mass Spectrometry Analysis of the *E. coli* Expressed Proteins

To confirm that the purified proteins are indeed the recombinant *Halobacterium *MCM proteins a matrix-assisted laser desorption ionization time-of-flight (MALDI-TOF) mass spectrometric (MS) analysis was used. MALDI-TOF MS was performed using an AB4700 Proteomics Analyzer (Applied Biosystems, Framingham, MA) using in-gel tryptic digestion of Coomassie-stained protein bands. Dry peptide samples were dissolved in 5 mg/ml α-cyano-4-hydroxy-cinnamic acid in 50% acetonitrile containing 0.1% trifluoroacetic acid, and manually spotted onto an ABI 01-192-6-AB target plate. MS-mode acquisitions consisted of 1,000 laser shots averaged over 20 sample positions. For MS/MS-mode acquisitions, 3,000 laser shots were averaged over 30 sample positions for PSD fragments. Automated combined acquisition of MS and MS/MS data was controlled with 4000 Series Explorer software 3.0. Data analysis was performed with GPS Explorer software 3.5 utilizing Mascot 2.0 (MatrixScience, London, UK) as the search engine. During searching, the mass tolerance was 0.08 Da for the precursor ions and 0.2 Da for the fragment ions. A protein was listed as identified protein when the MOWSE score was higher than the MOWSE score at which statistical significance (p < 0.05) occurred for that particular search.

### Generation of Antibodies

Urea purified MCM protein without intein was used to generate rabbit polyclonal antibodies by Cocalico Biologicals, Inc.

### Preparation of *Halobacterium* Cell Extract

Halobacterium sp. NRC-1 (ATCC number 700922) was grown in GN101 media (250g/L NaCl, 20g/L MgSO4, 2g/L KCl, 3g/L sodium citrate, 10g/L Oxoid brand bacteriological peptone) with the addition of 1 mL/L trace elements solution (31.5mg/L FeSO4·7H2O, 4.4mg/L ZnSO4·7H2O, 3.3mg/L MnSO4·H2O, 0.1mg/L CuSO4·5H2O) at 42°C with shaking at 220rpm. Beveled flasks were used to ensure proper oxygenation. Cultures were centrifuged at 8000 x g and a cell pellet from 25 ml of culture was resuspended in 1 ml of buffer containing 50 mM potassium phosphate (pH 7.0), 1 M NaCl, and 10% β-mercaptoethanol. The resuspended cells were sonicated on ice followed by centrifugation at 13,000 rpm for 10 min at 4ºC. The supernatant was kept at -20^o^C.

### Western Analysis

MCM protein with intein (20 ng) and without intein (1ng) and *Halobacterium *cell extract (0.5 μg and 1 μg) were separated on 10% SDS-PAGE. The gel was then electroblotted onto nitrocellulose BA83 membranes (Whatman) followed by Western analysis using 1:500 dilution of rabbit anti-MCM polyclonal antibodies and goat anti-rabbit antibody coupled to horseradish peroxidase (Epitomics) as secondary antibody. The blot was developed using ECL (GE healthcare) followed by exposure to X-ray film.

## RESULTS AND DISCUSSION

### Sequence Analysis of *Halobacterium* MCM Protein

**Fig. (1):**
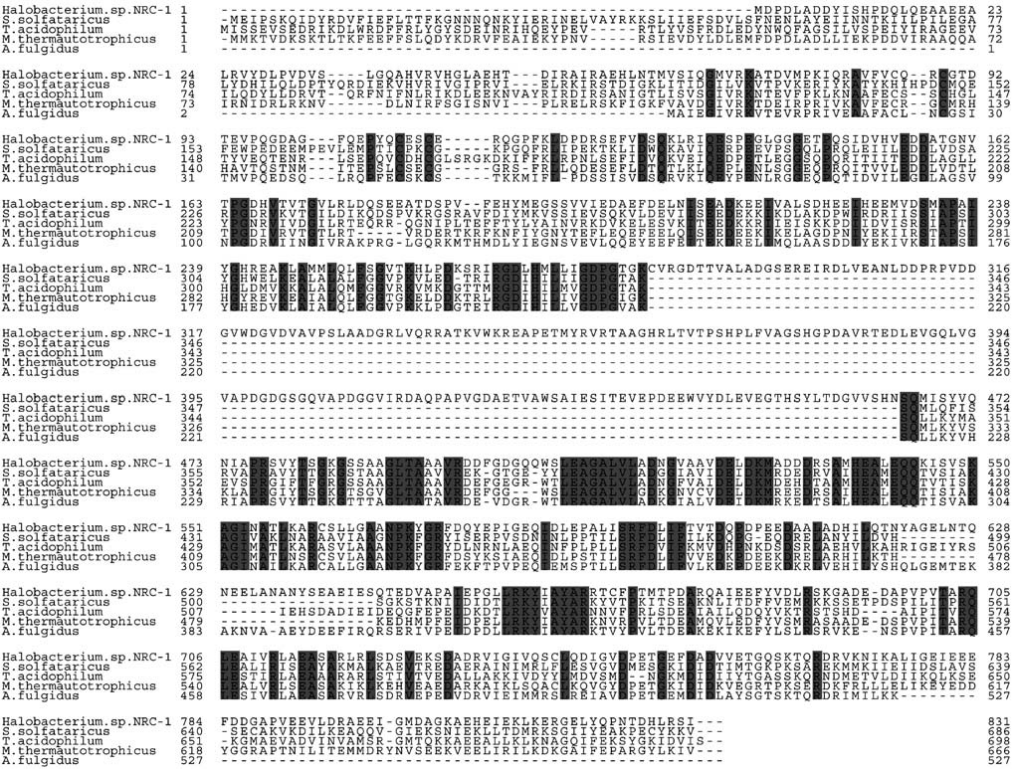
Alignment of full length *Halobacterium* MCM protein. Amino acid sequence alignment of five archaeal MCM helicases: *Halobacterium sp.* NRC-1, S. *solfataricus, T. acidophilum, M. thermautotrophicus,* and *A. fulgidus.* Highlighted residues are conserved in all five proteins. The long inserted sequence found in *Halobacterium* sp. NRC-1 is the intein.

Multiple alignment sequence analysis of archaealMCM proteins revealed that the *Halobacterium *protein contains a large insertion (Fig. **1**). Analysis of the insertion sequence and its integration site [5,6] clearly suggest that it is an intein. The intein is inserted in the middle of the Walker-A motif [7], a highly conserved motif which is found in all members of the AAA^+^ family of ATPases [8,9]. The sequence of the motif in *Halobacterium *MCM is GDPGTGKS and the intein is located between the highly conserved Lys and Ser residues.

The alignment also revealed that the C-terminal part of the MCM protein is more conserved than the N-terminal portion. The C-terminal part of the molecule contains the AAA^+^ catalytic domains, known to be highly conserved among different members of this family of enzymes. Nevertheless, the *Halobacterium *MCM as a whole clearly shows amino acid sequence similarity to other member of this family of helicases.

The sequence of the *Halobacterium *MCM also revealed that it contains a relatively small number of basic residues in comparison to other MCM proteins, and the ratio between negative and positive residues is much higher than in MCM proteins from non-halophilic archaea (Table **1**). Similar observations of charge distribution on proteins have also been observed when the entire proteome of *Halobacterium *was analyzed [10].

### Purification and Identification of *Halobacterium* MCM Helicase

**Fig. (2):**
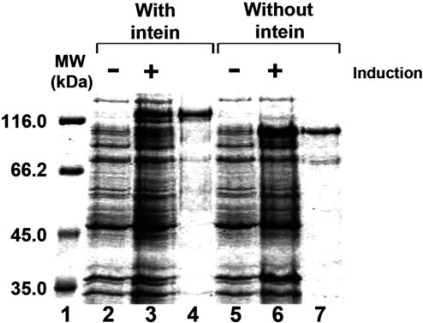
Purification of the *Halobacterium* MCM helicase. Uninduced and induced *E. coli* cells harboring pET-MCM (lanes 2 and 3) and pET-MCMint (lanes 5 and 6) and the purified MCM protein (0.5 µg) with intein (lane 4) and without intein (lane 7) were fractionated on 10% SDS-PAGE. Lane 1, molecular weight markers.

The MCM protein containing the intein was cloned into an *E. coli* expression vector and purified as described in “Material and Methods”. Although most of the proteins were not soluble, a small fraction was soluble and this fraction was purified (Fig. **2** lane 4). However, the intein is still present in the protein. It is known that in other intein-containing proteins expressed in *E. coli*, the intein is excised during induction prior to purification. The observation that the intein does not excise itself but stays as a part of the purified MCM suggests that either the *in vivo* conditions in *E. coli* (e.g. salt) are not favorable for the intein protease activity or that the protein is not properly folded.

In order to get a MCM protein without the intein, the intein was recombinantly removed from the gene and the expressed protein was purified as described in “Material and Methods” (Fig. **2** lane 7).

**Fig. (3):**
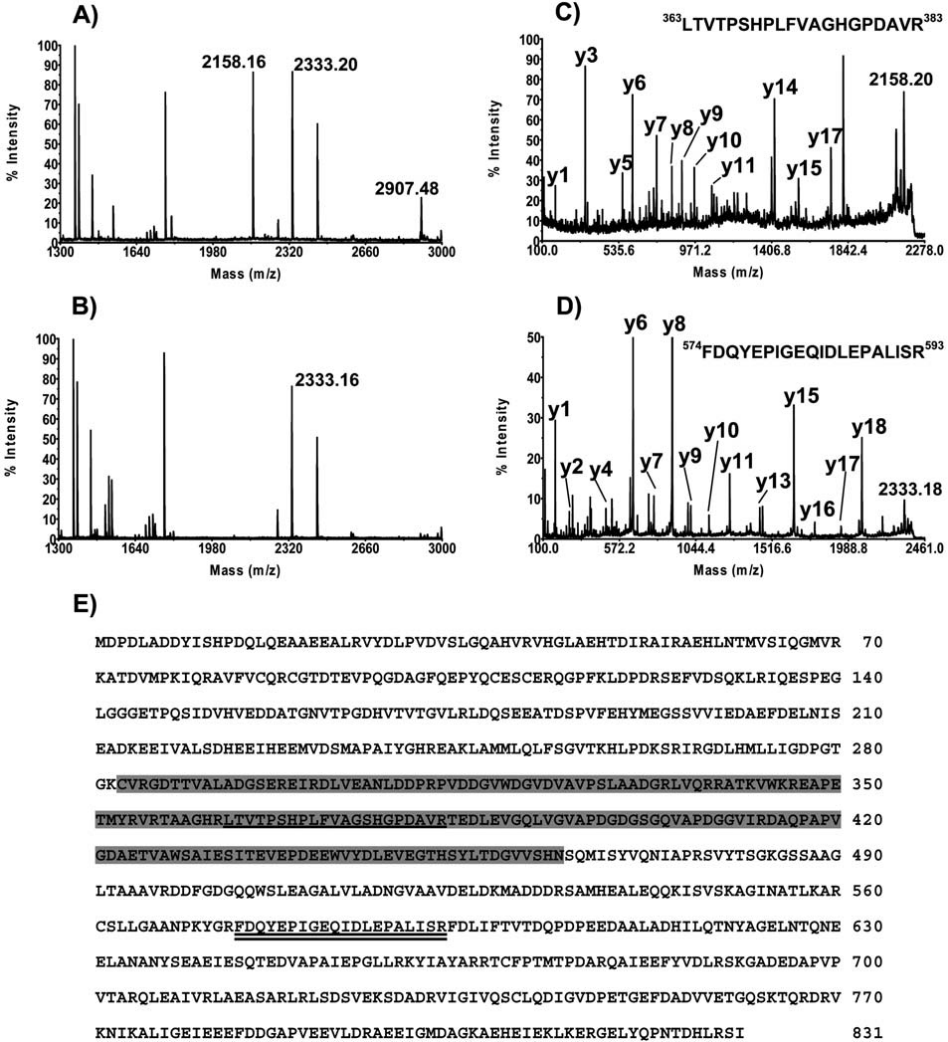
Mass spectroscopy analysis of the *E. coli* expressed proteins. MALDI-TOF spectra of *Halobacterium* MCM with **(A)** and without **(C)** intein were performed as described in “Material and Methods”. The 2158.16 and 2333.16 peaks were analyzed using MS/MS (**C** and **D**,respectively) as described in “Material and Methods”. Panel **E** shows the position of the peptide in the MCM amino acid sequence. Shaded areas show the location of the intein. The peptide corresponding to the 2158.16 peak is underlined and that for the 2333.16 peak is double underlined.

When analyzed on SDS-PAGE, both proteins migrate more slowly than predicted from their calculated molecular mass. The protein with intein migrates as a 123 kDa instead of 90 kDa molecule and the recombinant protein without intein migrates as 98 kDa instead of 71 kDa molecule. To confirm that the purified proteins are indeed the MCM proteins MALDI-TOF MS analysis was performed. The protein containing intein (Fig. **3A**) and without intein (Fig. **3B**) were analyzed. Both proteins contain peptides corresponding to the *Halobacterium *MCM proteins.

The peaks at m/z positions 2158.16 and 2907.48 are present only in the MCM protein containing the intein; (Fig. **3A**) they are absent in Fig. (**3B**). This suggests that these peaks originate from the MCM intein. A MS/MS analysis of the 2158.16 peak revealed that it is, indeed, the peptide from the intein sequence (Fig. **3C** and **3E**). Another peak, 2333.20, is found in both proteins (Fig. **3A** and **3B**). A MS/MS analysis of the peak showed that it is derived from a peptide located in the MCM protein but outside the intein (Fig. **3D** and **3E**). The combined results of the MS and MS/MS analysis recovered 43% and 50% of MCM protein with and without intein, respectively (data not shown). This suggests, with high confidence, that the two recombinant proteins isolated are the *Halobacterium *MCM protein.

### Detection of *Halobacterium* MCM Protein in vivo

As described above (Fig. **1**), the *Halobacterium *MCM gene encodes for a protein that contains an intein. As the intein is located in a highly conserved and important domain for helicase activity, it is presumed that, *in* *vivo*, the intein excises itself from the protein after translation and ligates the two other peptide fragments (exteins). Thus, the mature MCM protein will not contain the intein and will therefore be active [11]. It was therefore expected that in *Halobacterium *cells only the mature protein would be present. To determine if this is the case a western analysis was performed using cell extract and anti-MCM antibodies. As shown in Fig. (**4**) lanes 1 and 2, only the mature MCM, without the intein, could be detected.

## Figures and Tables

**Fig. (4):**
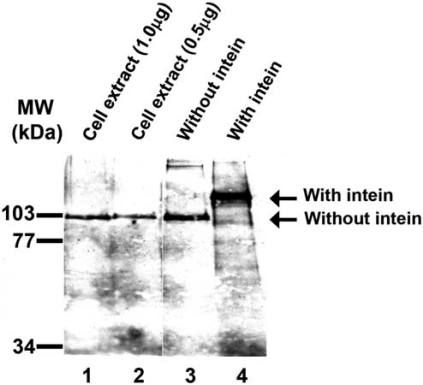
Only MCM protein without intein can be detected in *Halobacterium cells.* Western analysis of *Halobacterium* cell extract and recombinant MCM protein was performed as described in“Material and Methods”. Lane 1, cell extract (1 µg); lane 2, cell extract (0.5 µg); lane 3, purified MCM protein without intein (1ng); lane 4, purified full length MCM (20 ng). To the left are the positions of the molecular weight markers.

**Table 1:** Amino Acid Analysis of MCM Proteins

Organism	Length	KDa	Negativly charged: E+D (%)	Positively scharged: K+R (%)	Ratio: (E+D)/(K+R)	pI
***Halobacteriumsp.* NRC-1 with intein**	831	90.5	165 (19.9)	74 (8.9)	2.2	4.4
***Halobacteriumsp.* NRC-1 without intein**	649	71.2	130 (20.0)	59 (9.1)	2.2	4.4
***M. thermautotrophicus***	666	75.6	121 (18.2)	100 (15.0)	1.2	5.2
***T. acidophilum***	698	78.9	109 (15.6)	94 (13.5)	1.2	5.5
***S. solfataricus***	686	77.4	107 (15.6)	101 (14.7)	1.3	6
***A. fulgidus***	698	78.8	116 (16.6)	102 (14.6)	1.1	5.7
